# Should intravesical Bacillus Calmette–Guerin (BCG) treatment be administered to patients with T0 after repeat transurethral resection of bladder tumor in patients with high-risk non-muscle invasive bladder cancer?

**DOI:** 10.1371/journal.pone.0208267

**Published:** 2018-11-29

**Authors:** Hyeong Dong Yuk, Chang Wook Jeong, Cheol Kwak, Hyeon Hoe Kim, Ja Hyeon Ku

**Affiliations:** 1 Department of Urology, Inje University Sanggye Paik Hospital, Seoul, Korea; 2 Department of Urology, Seoul National University Hospital, Seoul, Korea; University of Oklahoma Health Sciences Center, UNITED STATES

## Abstract

We evaluated the effect of intravesical Bacillus Calmette–Guerin (BCG) and BCG maintenance therapy on the prognosis of patients with T0 after repeat transurethral resection of bladder mass (TURBT). This retrospective analysis involved 427 patients who underwent repeat TURBT within 6 weeks after initial TURBT from 2007 to 2016. Repeat TURBT was performed in patients with high-risk criteria. Patients who achieved T0 after repeat TURBT did or did not receive intravesical BCG therapy. Patients were divided into three groups: non-BCG, BCG induction, and BCG maintenance groups. The study included 106 patients who achieved T0 after repeat TURBT. The median follow-up was 63 months. There were no significant differences in T stage among the three groups. High grade ratio (p = 0.001) and concomitant CIS ratio (p = 0.037) were significantly higher in the BCG maintenance than in the other two groups. The recurrence rates in the non-BCG, BCG induction, and BCG maintenance groups were 46.2%, 28.3%, and 19.2%, respectively (p = 0.043). Recurrence-free survival was significantly higher in the BCG maintenance group than in the BCG induction group (p = 0.032). Progression-free survival was also higher in the BCG maintenance group than in the BCG induction group, but the difference was not significant (p = 0.056). Multivariate Cox regression analysis showed that only intravesical BCG maintenance therapy was significantly associated with recurrence (hazard ratio 0.016, p = 0.016). In high risk NMIBC patients, intravesical BCG maintenance treatment is required even at T0 after repeat TURBT. Intravesical BCG maintenance therapy of patients with T0 after TURBT reduces recurrence.

## Introduction

Bladder cancer is the eleventh most common cancer worldwide. Approximately 75% of newly diagnosed bladder cancer is non-muscle invasive bladder cancer (NMIBC) and most patients undergo transurethral resection of bladder mass (TURBT) as the first treatment[1]. TURBT is the standard treatment for NMIBC[2]. Tumor staging and grading are carried out clinically using TURBT, followed by additional treatment as needed[3]. However, in TURBT repeated after 1 to 6 weeks of initial TURBT, a residual tumor was detected in 26–83% of cases, and 9–49% showed clinical staging errors[4]. In another study, the likelihood of persistent residual tumor in T1 was 33–53%, and that of high-grade Ta was 41.4%. To reduce the risk of understaging, a few guidelines recommend repeat TURBT as needed. Most of the targets for repeat TURBT include high-grade Ta and T1(2, 5). The guidelines recommend repeat TURBT in all T1 tumors and high-grade tumors except low-grade Ta and primary carcinoma in situ (CIS), In addition, repeat TURBT is recommended in the absence of muscle in the specimen after the initial resection and incomplete initial TURBT (2, 5). Most patients undergoing repeat TURBT belong to high-risk NMIBC category, and most of them are candidates for intravesical Bacillus Calmette–Guerin (BCG) therapy[3]. In high-risk NMIBC patients, intravesical BCG treatment has been reported to reduce recurrence and prevent or delay disease progression[5, 6]. Thus, repeat TURBT and intravesical BCG treatment are important treatment options for high-risk NMIBC. However, the follow-up treatment according to pathologic results after repeat TURBT is not well established. In particular, the role of observation versus intravesical BCG treatment in cases of T0 after repeat TURBT is not clear. In this study, we investigated the need for intravesical BCG treatment at T0 after repeat TURBT and discussed the appropriate method.

## Materials and methods

### Ethics statement

The study was approved by the Institutional Review Board of the Seoul National University Hospital Biomedical research institute (IRB No. H-11712-059-905). We conducted a retrospective case study and were exempted from obtaining prior consent of the patients. The research protocol was in accordance with the Declaration of Helsinki guidelines.

### Study sample

We reviewed medical records of 427 patients who underwent repeat TURBT at the Seoul National University Hospital from 2007 to 2016. Among these patients, 106 with T0 after repeat TURBT were finally included in the study. All the patients were diagnosed as high-risk NMIBC after initial TURBT and subjected to repeat TURBT.

### Study design

We divided the patients into three groups according to the intravesical BCG intervention: non-BCG BCG induction and BCG maintenance. The first group included the group that was not treated with intravesical BCG for T0 following repeat TURBT. The second group was exposed to intravesical BCG with only six induction treatments performed during the first 6 weeks. The third group involved patients undergoing intravesical BCG treatment followed by maintenance according to the guidelines. Pathologic findings of T0 after repeat TURBT were confirmed by two experienced genitourinary pathologists. The TNM stage and tumor grade were determined according to the 2010 American Joint Committee on Cancer classification and the 2004 World Health Organization/International Society of Urologic Pathology consensus classifications. We investigated patient and clinical demographics: age, sex, height, weight, body mass index (BMI), gross hematuria, pathologic T stage, tumor grade, presence of carcinoma in situ (CIS), lymphovascular invasion (LVI), number of tumors and tumor size. We also investigated the oncologic outcomes: radical cystectomy, recurrence, recurrence at the upper urinary tract, progression and mortality.

In the absence of recurrence, progression, or metastasis, a follow-up was performed every 3 months until 3 years, followed every 6 months from year 5, and every 1 year thereafter. Routine blood and urine tests, urine cytology, and cystoscopy were performed at every follow-up. CT urography was performed every year, and LDCT, MR urography, bone scan, and PET-CT were performed if necessary.

The induction group was subjected to treatment only 6 times, once every week. BCG maintenance treatment was administered according to the Southwest Oncology Group (SWOG) protocol. The maintenance group was treated for up to three years according to the prescribed protocol.

### Statistical analysis

The primary end point was the recurrence-free survival (RFS). The secondary end points were progression-free survival (PFS) and overall survival (OS). The interpretation of the results was based on statistical analysis. The mean and standard deviation (SD) were used for the continuous variables, and the median and the interquartile range (IQR) were used for a few variables. ANOVA was used for comparison between groups. The nominal variables used frequency of occurrence and percentage. Statistical significance was considered when the p-value was less than 0.05. In the analysis of oncological outcomes, Kaplan-Meier survival analysis and log rank test were used to compare groups, and various prognostic factors were analyzed by Cox proportional hazards regression model.

The statistical program used IBM SPSS Statistics version 22.0 (IBM, Armonk, New York, USA).

## Results

### Clinical and oncological characteristics of patients

As shown in Table 1, the clinical and pathological characteristics and oncological outcomes of the patients included in this study are listed. The median follow-up was 61 months (IQR 21–108), and more than 80% of all patients were male, with 50–70% manifesting preoperative gross hematuria symptoms. In the patients who underwent intravesical BCG treatment, the proportion of high-grade tumors was high with an increased rate of accompanying CIS. Other pathologic characteristics were not significantly different. The BCG maintenance group had a significantly lower recurrence rate of oncological outcomes (p = 0.043).

**Table 1:** Clinical and oncological characteristics of patients.

Clinical characteristics	Non-BCG group	BCG induction	BCG maintenance	P- value
Number of patients	52	29	26	
Age (mean±SD)	61.9 ± 10.2	65.4 ± 7.2	68.0 ± 12.9	
BMI (kg/m^2^)	24.06 ± 2.91	24.38 ± 2.28	24.05 ± 23.20	
Gender				0.833
Male	44(84.6%)	24(82.8%)	23(88.5%)	
Female	8(15.4%)	5(17.2%)	3(11.5%)	
GHU	37(71.2%)	20(69.0%)	13(50.0%)	0.273
T stage				0.052
Ta	4(7.7%)	3(10.3%)	7(26.9%)	
T1	48(92.3%)	26(89.7%)	19(73.1%)	
Tumor grade				0.001
Low	14(26.9%)	0(0%)	1(3.8%)	
High	38(73.1%)	29(100%)	25(96.2%)	
Concomitant CIS	0(0%)	5(17.2%)	4(15.4%)	0.037
LVI	0(0%)	2(6.9%)	0(0%)	0.169
Tumor size				0.788
< 3cm	37(71.2%)	19(65.5%)	17(65.4%)	
≥ 3cm	13(25.0%)	9(31.0%)	9(34.6%)	
Tumor multiplicity				0.715
1	29(55.8%)	15(51.7%)	16(61.5%)	
2–7	17(32.7%)	10(34.5%)	10(38.5%)	
>8	6(11.5%)	4(13.8%)	0(0%)	
Radical Cystectomy	6(11.5%)	6(20.7%)	2(7.7%)	0.325
Recurrence	24(46.2%)	14(48.3%)	5(19.2%)	0.043
Upper tract recurrence	2(3.8%)	1(3.4%)	0(0%)	0.504

Abbreviations: BCG, bacillus Calmette-Guerin; BMI, body mass index; CIS, carcinoma in situ; LVI, lymphovascular invasion;

### Association of intravesical BCG treatment with oncological outcomes

**Fig 1:**
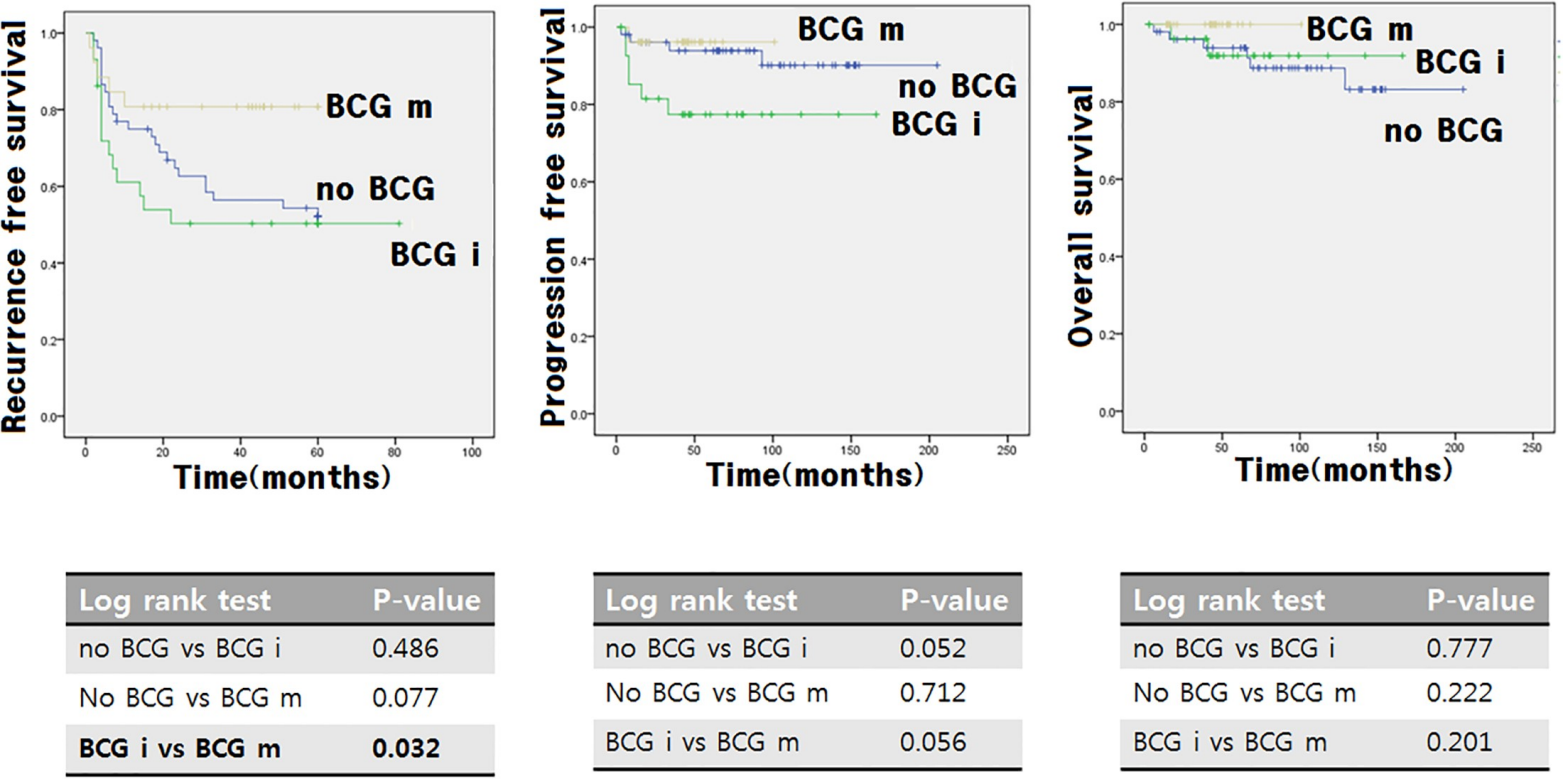
Kaplan-Meier survival curves of oncological outcomes in patients treated with intravesical BCG following repeat treansurethral resection of bladder mass.

Fig 1 shows the comparative effect of intravesical BCG treatment on oncological outcomes in pT0 patients with repeat TURBT in high-risk NMIBC patients in different groups. In the B and C graphs of Fig 1, we found no difference in PFS across the different groups. However, the graph A suggests good prognosis in terms of RFS in the BCG maintenance group compared with the BCG induction group.

### Significant predictors of recurrence-free survival by Cox multivariate regression analysis in the overall population

We performed a Cox regression analysis of RFS predictors in pT0 patients with repeat TURBT in high-risk NMIBC patients (Table 2). Important factors predicting RFS after repeat TURBT include T1 stage and BCG treatment. BCG maintenance methods were significant predictors of RFS (HR 0.198; 95% CI = 0.051–0.019).

**Table 2:** Multivariate Cox proportional hazard ratio analysis of predictive factors for recurrence-free survival.

parameter	Univariate analysis		Multivariate analysis	
	HR (95% Cl)	P-value	HR (95% Cl)	P-value
Age	0.984 (0.951–1.024)	0.472	0.972 (0.926–1.021)	0.254
Gender	1.577 (0.506–4.914)	0.432	0.175 (0.028–1.098)	0.063
T stage				
Ta	reference		reference	
T1	3.325 (1.104–5.365)	0.008	3.323 (1.232–5.271)	0.003
Grade				
Low	reference		reference	
High	0.991 (0.325–3.019)	0.987	0.632 (0.204–1.951)	0.424
CIS	1.379 (0.326–5.841)	0.662	0.777 (0.107–5.638)	0.062
LVI	0.667 (0.041–10.954)	0.776	1.871 (0.094–37.059)	0.681
Tumor multiplicity		0.078		0.136
1	reference		reference	
2–7	0.266 (0.070–1.013)	0.052	0.091 (0.011–0.785)	0.029
>8	0.538 (0.132–2.193)	0.387	0.230 (0.028–1.880)	0.170
Tumor size				
< 3cm	reference		reference	
≥ 3cm	1.105 (0.467–2.611)	0.821	1.165 (0.425–2.884)	0.954
BCG instillation		0.054		0.047
Non-BCG	reference		reference	
BCG induction	1.577 (0.667–3.731)	0.300	0.866 (0.275–2.729)	0.806
BCG maintenance	0.026 (0.093–0.784)	0.016	0.198 (0.051–0.763)	0.019

Abbreviations: BCG, bacillus Calmette-Guerin; CIS, carcinoma in situ; LVI, lymphovascular invasion;

## Discussion

International guidelines recommend intravesical BCG as an adjuvant treatment in patients with intermediate-to-high risk NMIBC[2]. Several studies have reported that BCG intravesical treatment after TURBT lowers the recurrence rate in NMIBC rather than in TURBT alone or TURBT with chemotherapy[5]. In the EORTC study, BCG maintenance therapy reported a 27% reduction in disease progression. In addition, EORTC reported that a 3-year maintenance therapy with intravesical BCG reduced the recurrence rate compared to the 1-year maintenance therapy in high-risk NMIBC patients[7]. Intravesical BCG induction therapy was first introduced by Morales in 1976. He explained that adjuvant intravesical BCG treatment lowered the recurrence rate[8]. Since then, several studies investigated the schedule of maintenance[9–13]. Although some studies failed to demonstrate the efficacy of maintenance, most studies have reported that maintenance therapy for more than one year prevents recurrence and progression[14].

This paper discusses the role of BCG treatment after repeat TURBT in high-risk NMIBC patients. This study also confirmed prognostic factors associated with recurrence, progression, and overall survival.

We found a higher number of high-grade patients in the BCG groups than in the non-BCG group. In addition, there were more CIS patients in the BCG groups than in the non-BCG group. High-grade and CIS rates showed a significant increase in the BCG groups than in the observation group (Table 1). Patients who have a relatively higher risk may have been exposed to additional intravesical BCG treatment. The highest group of T1HG + CIS patients was found to have received 100% BCG treatment. In other words, these higher-risk patients received intravesical BCG treatment. However, patients with BCG maintenance have the lowest recurrence rate. Although not significant, the rate of radical cystectomy was the lowest in the BCG maintenance group. We compared OS, RFS, and PFS between non-BCG, BCG induction, and BCG maintenance groups (Fig 1). In the RFS graph, the BCG maintenance group showed a significantly better prognosis compared with the BCG induction group. In univariate and multivariate analyses of risk factors for RFS, the BCG maintenance therapy (HR 0.278, IQR 0.091–0.849, P = 0.050) was the only significant factor (Table 2). Although repeat TURBT patients were not included in the study, RCTs reported differences in prognosis according to BCG duration[15]. Clinical trials of SWOG reported a significantly better prognosis in the maintenance group compared with the control group only with induction. The RFS was 76.8 months in the maintenance arm and 35.7 months in the control arm.[13] In the other RCT, the two-year RFS was higher in the maintenance group than in the induction group (95.8% vs. 74.1%). In univariate analysis, the maintenance treatment played a significant role in recurrence[16]. In another RCT, the two-year RFS in the maintenance group was 84.6% and 65.4% higher than in the induction group, and the RFS after TURBT was significantly longer in the maintenance group than in the induction group [17]. In previous studies, dose and maintenance therapy of intravesical BCG treatment resulted in significant benefits in reducing the recurrence rate after TURBT. However, there were no significant differences in other oncologic outcomes such as PFS, OS and cancer-specific survival (CSS)[15, 18, 19] similar to our study results. BCG maintenance treatment was significantly associated with RFS, although the patients were different.

Our research has some limitations. First, a retrospective study involving single tertiary institution is not free from selection bias. In addition, this could undermine the association between BCG and RFS. The size of the patient population was small. Although few patients showed T0 status after repeat TURBT, it was a relatively small sample size. Better evidence based on a prospective study or RCT involving large number of patients is needed. Despite the several limitations, the study has important clinical implications in the absence of a previous study that provided clear-cut evidence supporting BCG treatment maintenance therapy in patients with T0 after repeat TURBT.

## Conclusion

Intravesical BCG maintenance treatment is required even at T0 after repeat TURBT in high risk NMIBC patients. Intravesical BCG maintenance therapy of patients with T0 after TURBT reduces recurrence.
